# Supplementary material for: GUNC: detection of chimerism and contamination in prokaryotic genomes
Source: Genome Biol. 2021 Jun 13;22:178. doi: 10.1186/s13059-021-02393-0 (PMC8201837; doi:10.1186/s13059-021-02393-0)
Supplement: Supplementary file 1 — Additional file 1: Figure S1. a Percent stacked bar chart of CheckM inferred marker lineage levels (colors) for type 3a simulated chimeric genomes (see Methods & Fig. 2a) across different: 1) divergence levels of source genomes (x-axis); 2) simulated portions of contamination (columns); and 3) scenarios of contamination (‘added’ vs ‘replaced’, rows; see Methods). In a and b, the first column (“0”) are clean (non-chimeric) genomes shown for comparison. b Average inferred CheckM marker lineage depth (y-axis) of simulated chimeric genomes under different contamination scenarios (‘added’ in dark blue; ‘replaced’ in light blue). The true taxonomic depth of divergence between source genomes are indicated in green. c Equivalent to a, but using chimeric genomes simulated from multiple sources (type 3b in Fig. 2a). Columns indicate the number of equally contributing source genomes (n_sources); rows indicate simulation setups (‘0.5’ if 50% of each source genome was used; ‘1/n_sources’ for equal source parts; see Methods). In c & d, the first column (“1”) are clean (non-chimeric) genomes, the second column (“2”) are type 3a genomes as in a & b, shown for comparison. d Average inferred CheckM marker lineage depths (y-axis) with different portions of contamination, equivalent to panel b. Figure S2. Comparison of median scores from GUNC and CheckM of simulations of genomes type 3a and 3b where source genomes make equal contributions summing 1 in total (e.g. 0.2 from each of 5 sources or 0.25 from each of 4 sources). This shows that the trend from Fig. 2b persists when multiple source genomes are mixed in a simulated chimeric genome. Figure S3. F-scores of distinction between clean and chimeric genomes across all divergence levels of source genomes for different simulation scenarios. MIMAG medium is CheckM contamination < 10% and CheckM completeness ≥50%. MIMAG high is CheckM contamination <5% and CheckM completeness >90% and due to irrelevance to our simulations we decided that a [file 13059_2021_2393_MOESM1_ESM.pdf]

Supplementary Figures (Additional file 1)

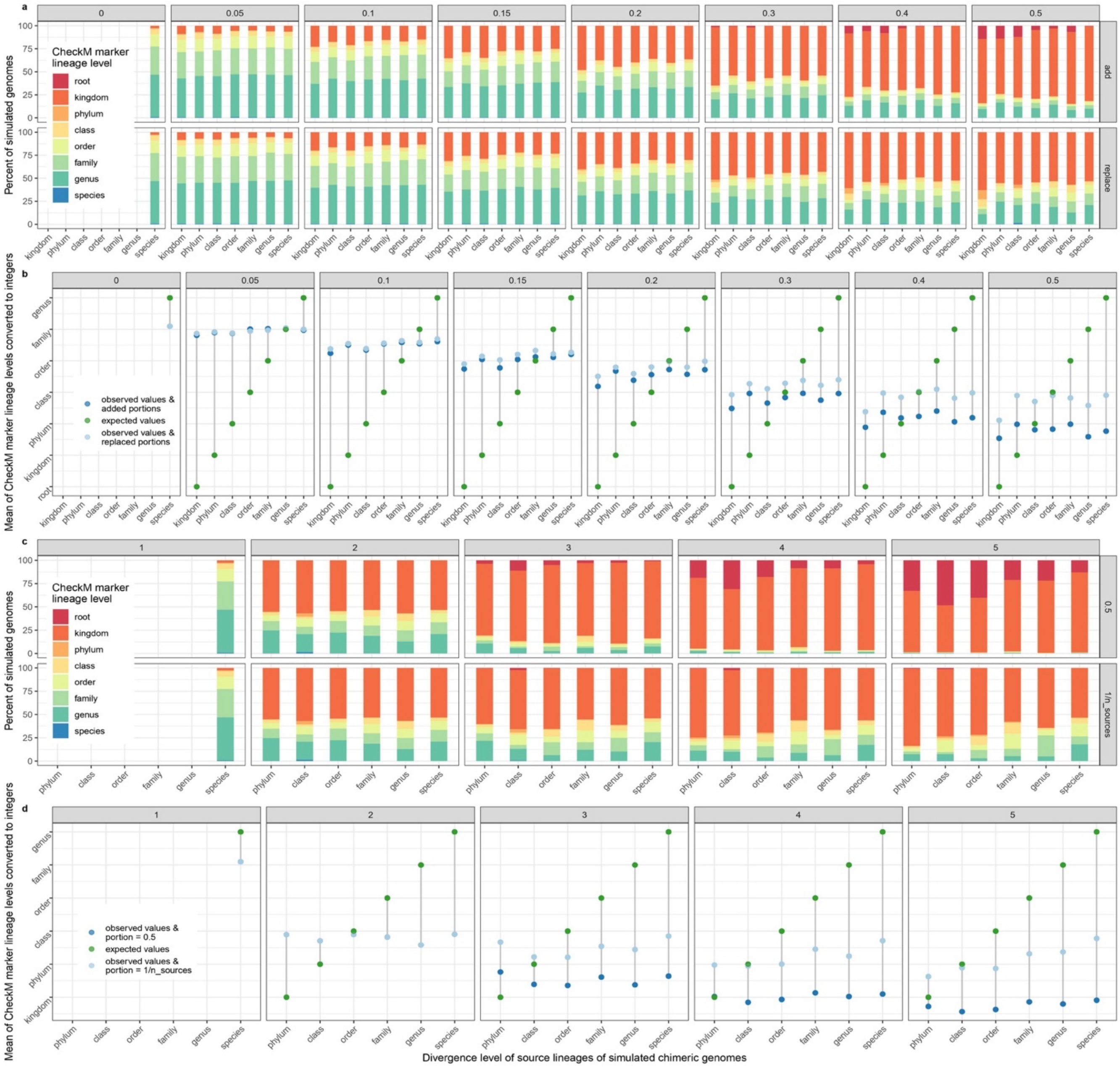

Fig. S1. **a** Percent stacked bar chart of CheckM inferred marker lineage levels (colors) for type 3a simulated chimeric genomes (see Methods & Fig. 2a) across different: 1) divergence levels of source genomes (x-axis); 2) simulated portions of contamination (columns); and 3) scenarios of contamination ('added' vs 'replaced', rows; see Methods). In a and b, the first column ("0") are clean (non-chimeric) genomes shown for comparison. **b** Average inferred CheckM marker lineage depth (y-axis) of simulated chimeric genomes under different contamination scenarios ('added' in dark blue; 'replaced' in light blue). The true taxonomic depth of divergence between source genomes are indicated in green. **c** Equivalent to a, but using chimeric genomes simulated from multiple sources (type 3b in Fig 2a). Columns indicate the number of equally contributing source genomes (n_sources); rows indicate simulation setups ('0.5' if 50% of each source genome was used; '1/n_sources' for equal source parts; see Methods). In c & d, the first column ("1") are clean (non-chimeric) genomes, the second column ("2") are type 3a genomes as in a & b, shown for comparison. **d** Average inferred CheckM marker lineage depths (y-axis) with different portions of contamination, equivalent to panel b.

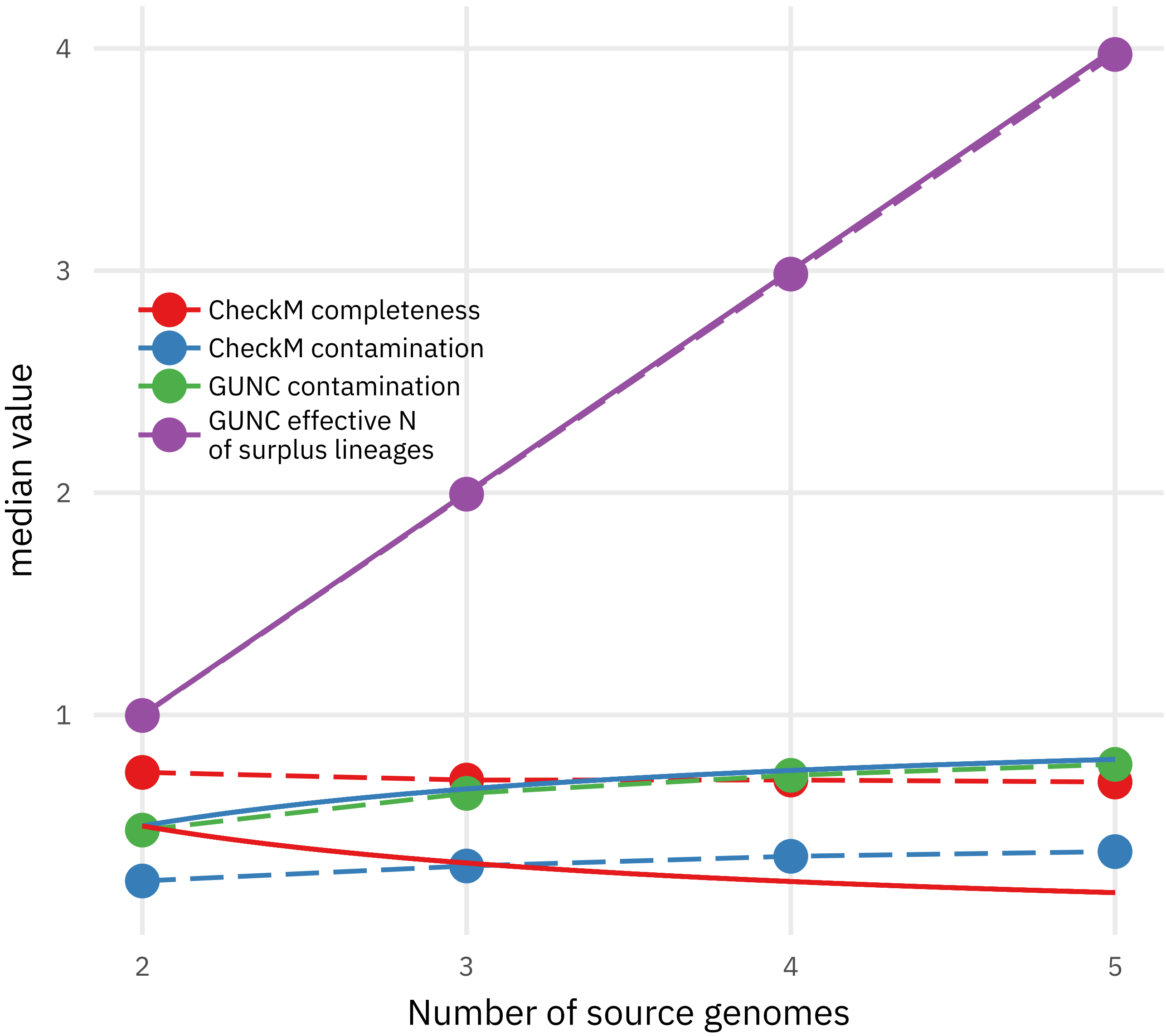

Fig. S2. Comparison of median scores from GUNC and CheckM of simulations of genomes type 3a and 3b where source genomes make equal contributions summing 1 in total (e.g. 0.2 from each of 5 sources or 0.25 from each of 4 sources). This shows that the trend from Fig. 2b persists when multiple source genomes are mixed in a simulated chimeric genome.

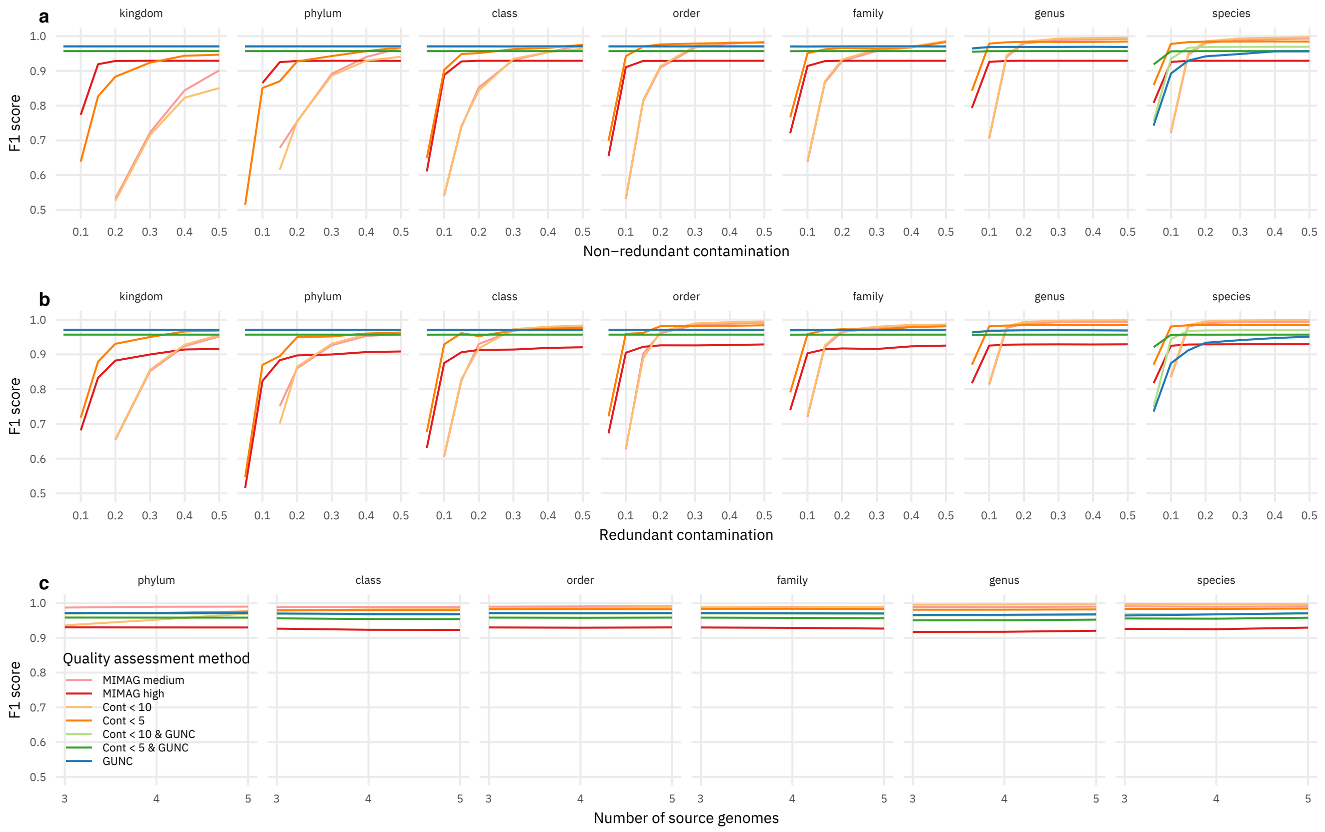

Fig. S3. F-scores of distinction between clean and chimeric genomes across all divergence levels of source genomes for different simulation scenarios. MIMAG medium is CheckM contamination < 10% and CheckM completeness >50%. MIMAG high is CheckM contamination <5% and CheckM completeness >90% and due to irrelevance to our simulations we decided that additional criteria of presence of rRNAs and tRNAs can be ignored here. “Cont” stands for CheckM contamination and GUNC means GUNC CSS of <0.45 or GUNC contamination <2%. a type 3a non-redundant contamination. b type 3b redundant contamination. c type 3b.

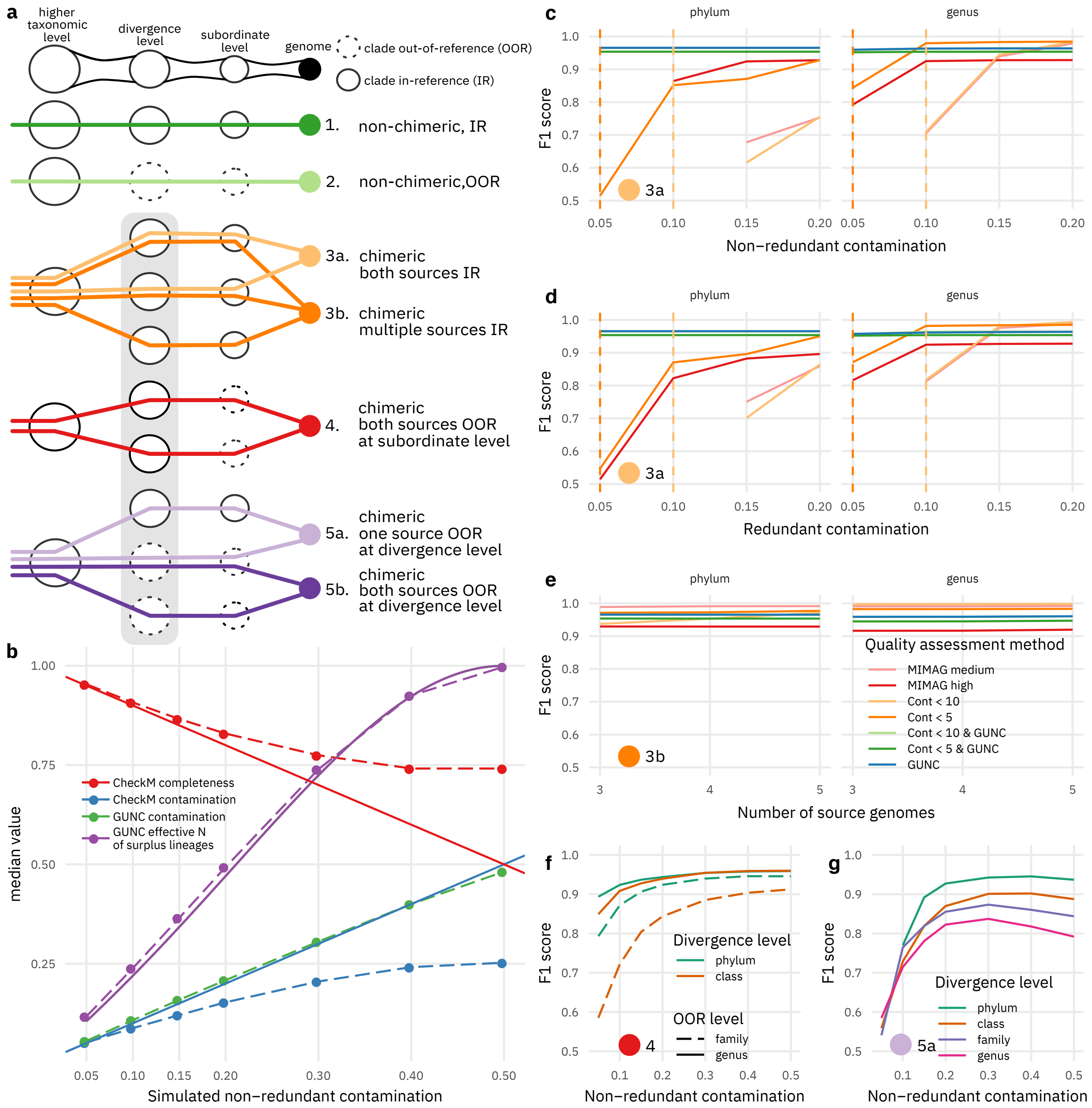

Fig. S4. Equivalent to Fig 2, but using inferred GTDB taxonomy for GUNC's default reference DB instead of NCBI taxonomy (default reference genomes with GTDB taxonomy).

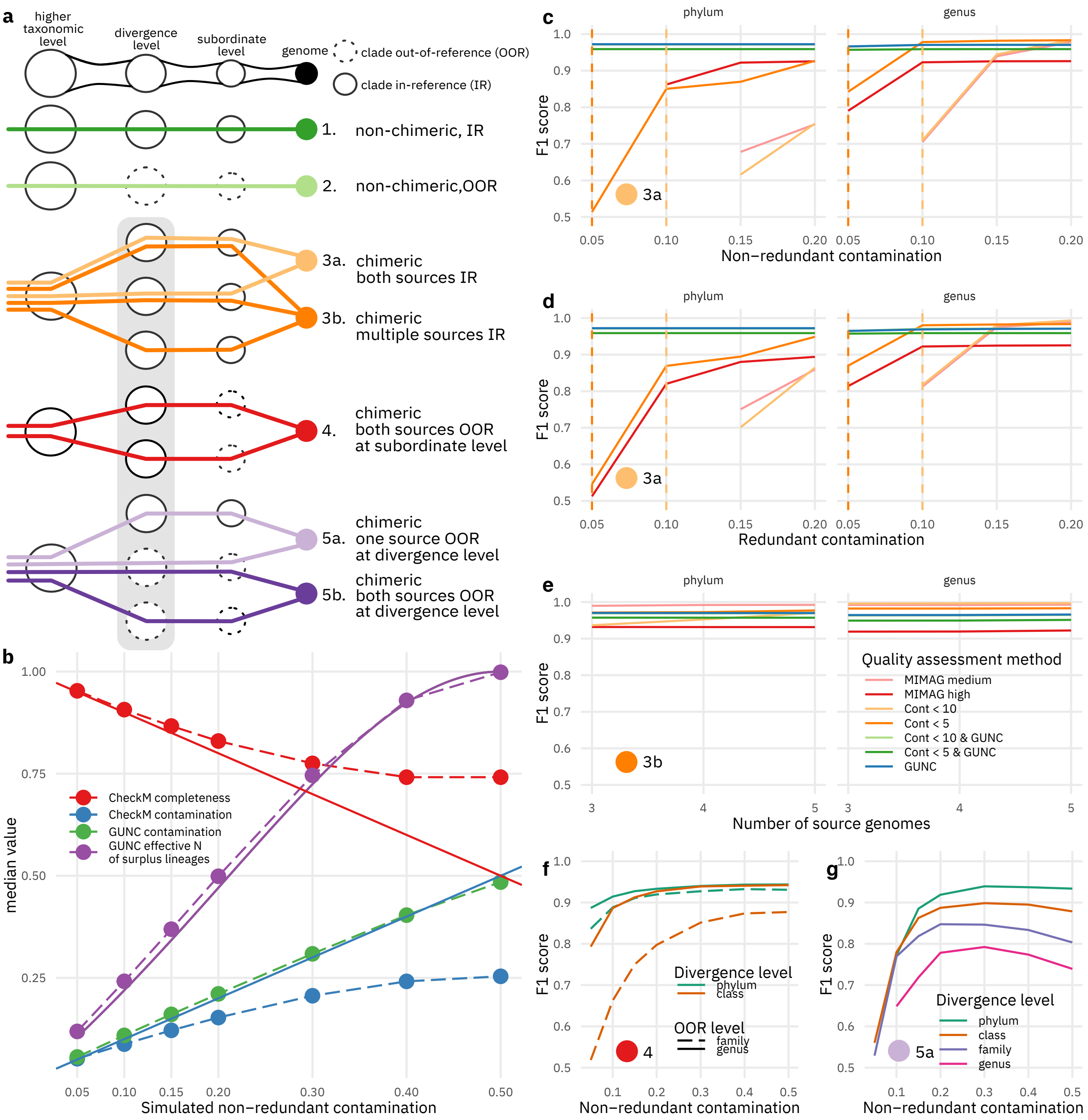

Fig. S5. Equivalent to Fig 2, but using an alternative GTDB-based GUNC reference DB (GTDB v95 genomes and GTDB taxonomy).

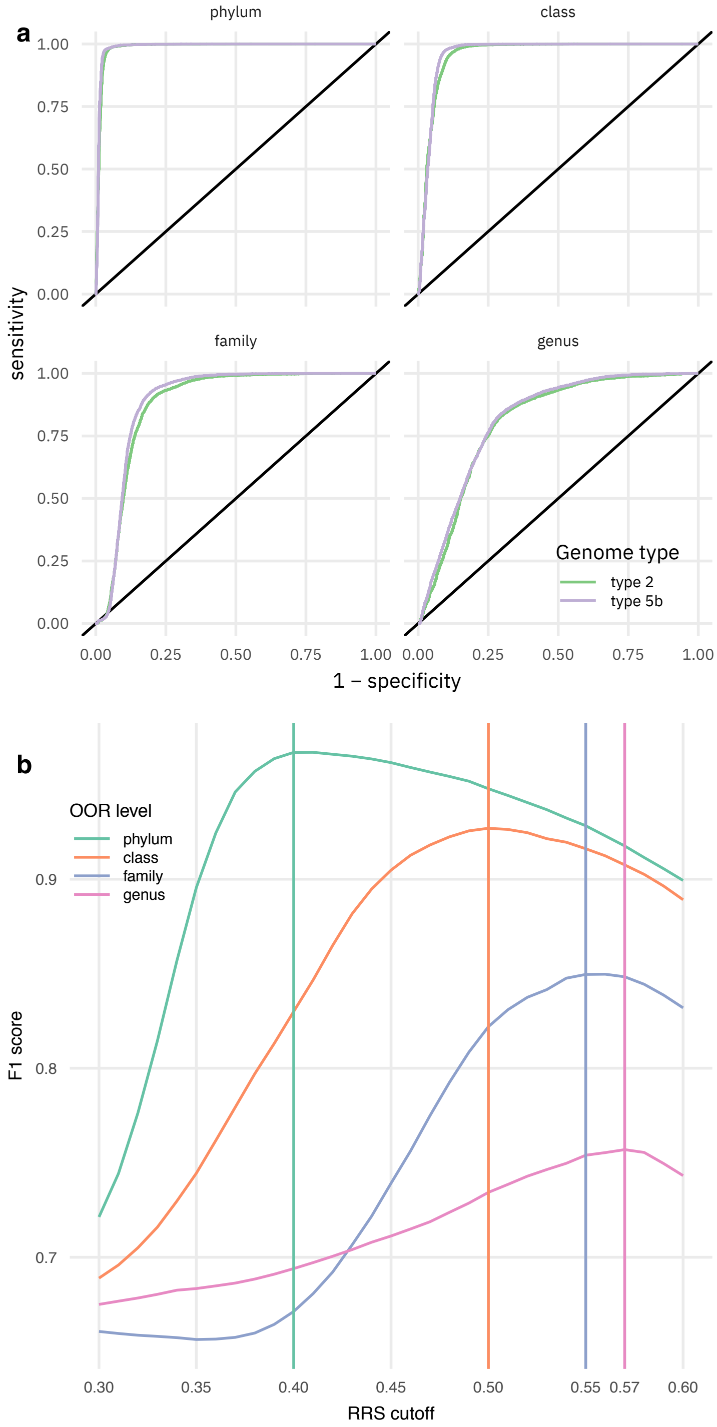

Fig. S6. **a** ROC-curves and AUCs of separation of genomes in-reference (type 1) from genomes out-of-reference (types 2 and 5b) at different out-of-reference levels (faceting) using GUNC reference representation scores (RRS) at matching taxonomic levels. **b** F1-scores (y-axis) of separation of types 2 & 5a from type1 across different RRS cutoffs (x-axis) different out-of-reference (OOR) levels (colors) at which these genomes have no reference representation. GUNC scores at the taxonomic level identical to OOR level were used. Vertical lines indicate RRS scores with highest F1-scores at each OOR level. Cutoff of RRS <0.5 is used to label genomes as "OOR" irrespective of the taxonomic level of max CSS.

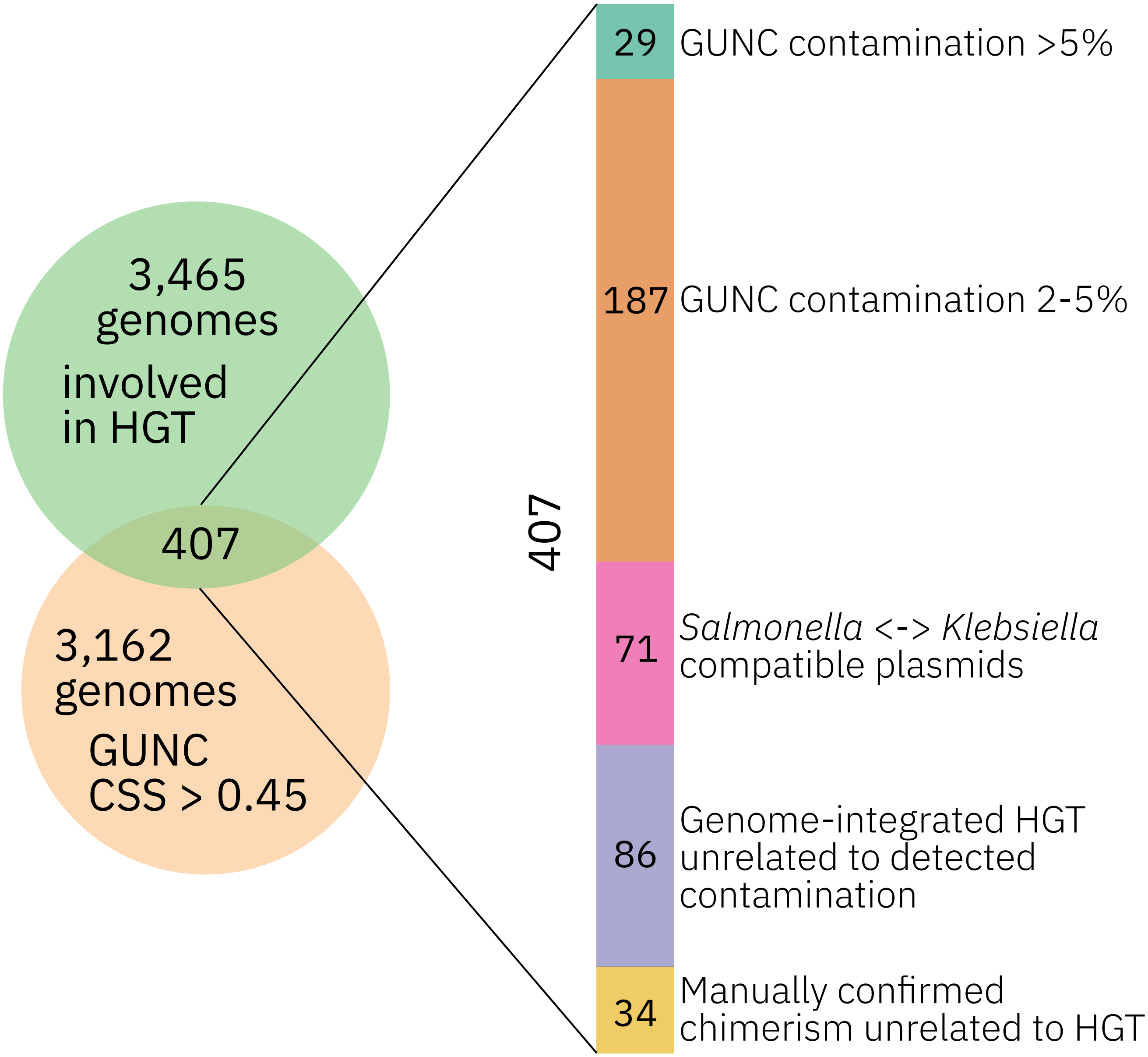

Fig. S7. The Venn diagram on the left illustrates the overlap (407 genomes) between sets of genomes associated with HGT (3,465 genomes) and those potentially chimeric (3,162 genomes) according to GUNC (CSS >0.45 & contamination >2%). The stacked bar plot on the right indicates the numbers of genomes from the overlap in each category. These categories do have overlaps and therefore genomes in them were counted and removed from the set used to count remaining categories in the following order of their genome counts: 71 > 34 > 86 > 187 & 29.

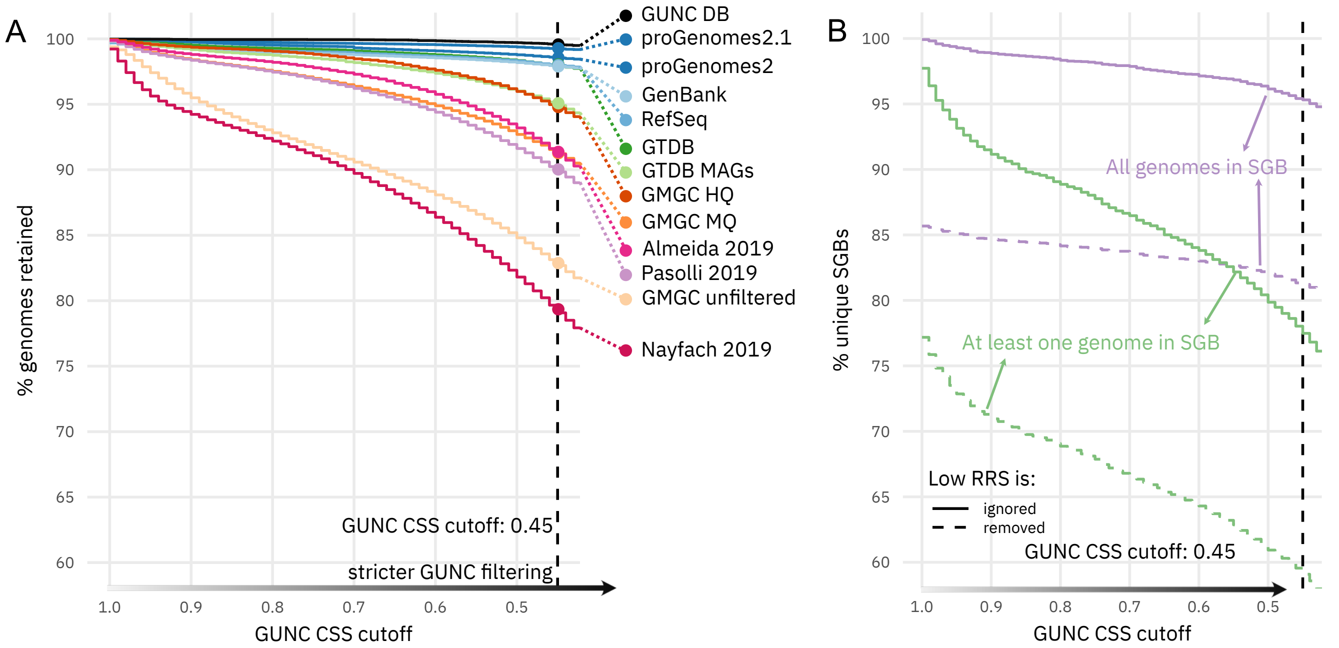

Fig. S8. **a** Cumulative plot summarizing genome qualities of various sets of genomes represented by lines of different colors. Any point in a plot indicates a portion of genomes retained in a set (y-axis) after filtering out genomes with GUNC CSS higher than the cutoff (x-axis) & GUNC contamination >5% (ignoring species level scores). **b** Cumulative plot illustrating the number of species-level genome bins (SGBs) (from Pasolli et al. 2019). Lines indicate the portion of unique SGBs retained (y-axis) after filtering out SGBs where either "all" or "at least one" genome has GUNC CSS score higher than the cutoff (x-axis) & GUNC contamination >5%.

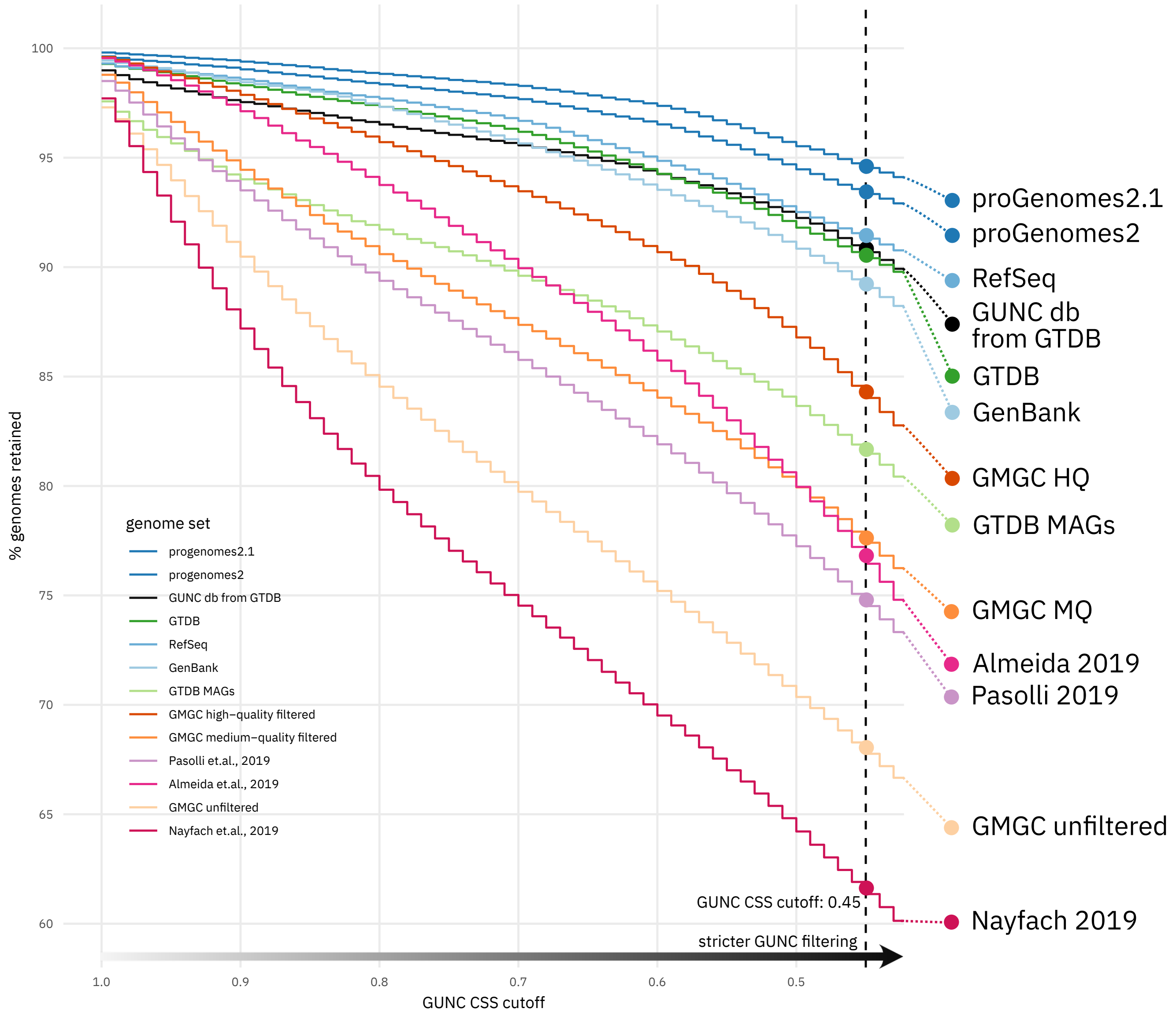

Fig. S9. Cumulative plots summarizing genome quality for various genome reference and MAG datasets. This plot is equivalent to main figure 3a, but using a reference set based on GTDB v95 instead of GUNC's default based on proGenomes 2.1 (see Methods for details). Note that the Almeida, Pasolli and Nayfach sets were pre-filtered using variations of the MIMAG medium criterion based on CheckM estimates. GTDB, Genome Taxonomy Database; GMGC, Global Microbial Gene Catalogue.

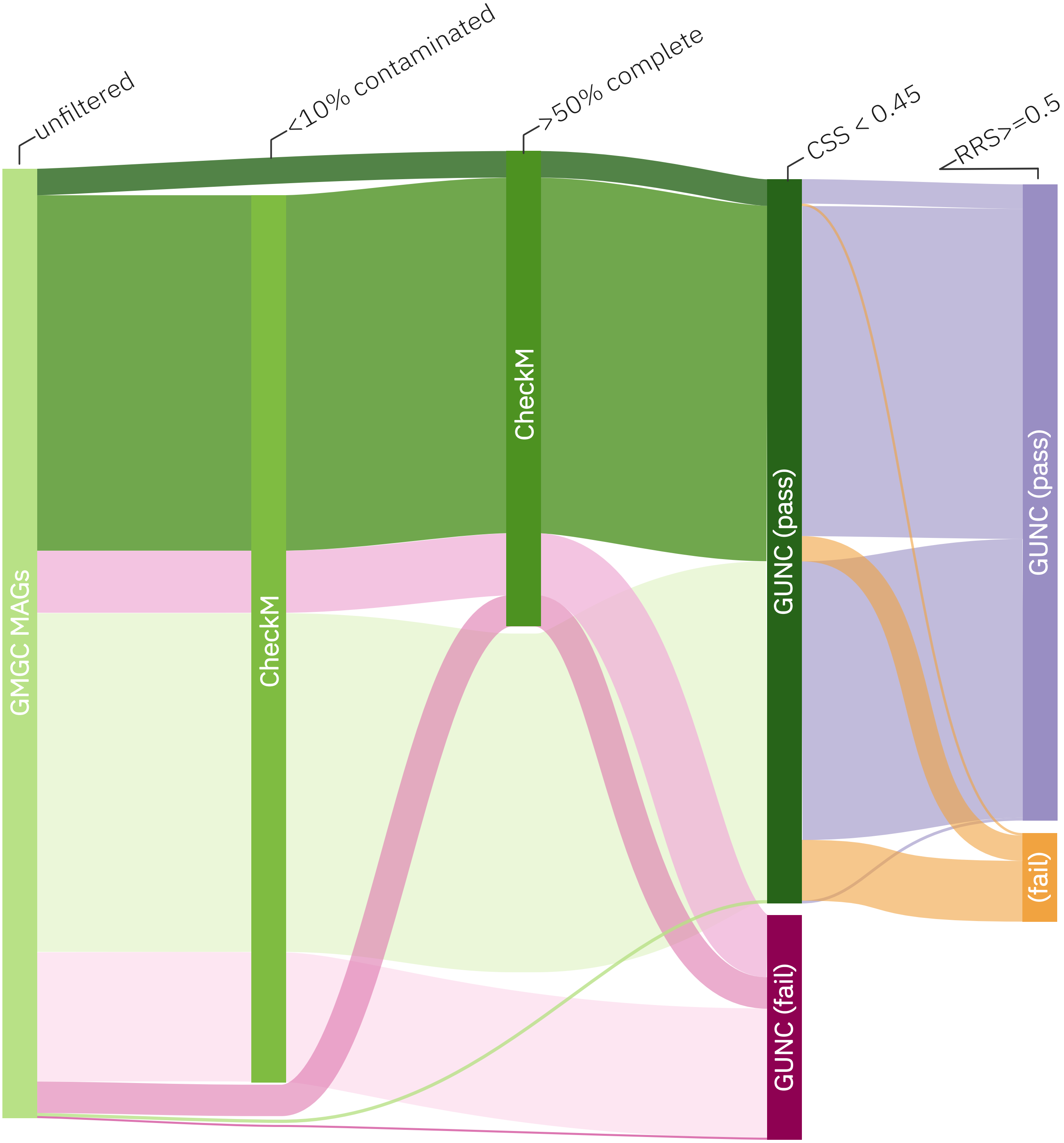

Fig. S10. Alluvial illustration of the fate of genomes in GMGC based on filters by GUNC and CheckM. Three filters are: 1) CheckM contamination <10%; 2) CheckM completeness <50%; 3) GUNC CSS <0.45 or GUNC contamination <2% (ignoring species level scores). The illustration shows the orthogonality and complementarity between GUNC and CheckM filters.

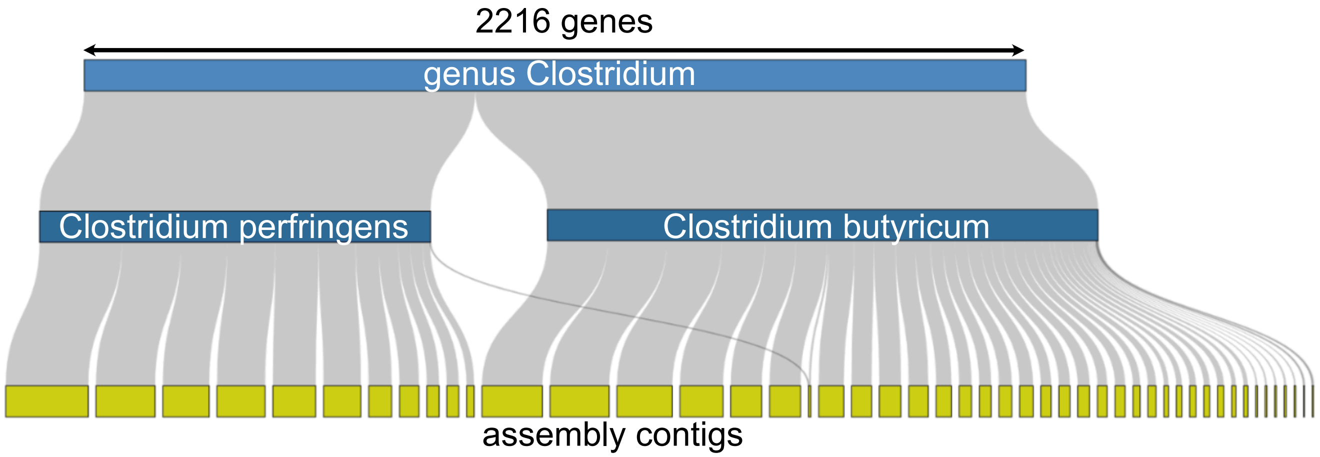

Fig. S11. Alluvial illustration of MAG “SRR1779121_bin.6” from Almeida et.al. 2019 that shows that GUNC can detect chimerism of related species when both source species have reference representation. The CheckM completeness is 79.31 and contamination is 1.72 for this MAG.

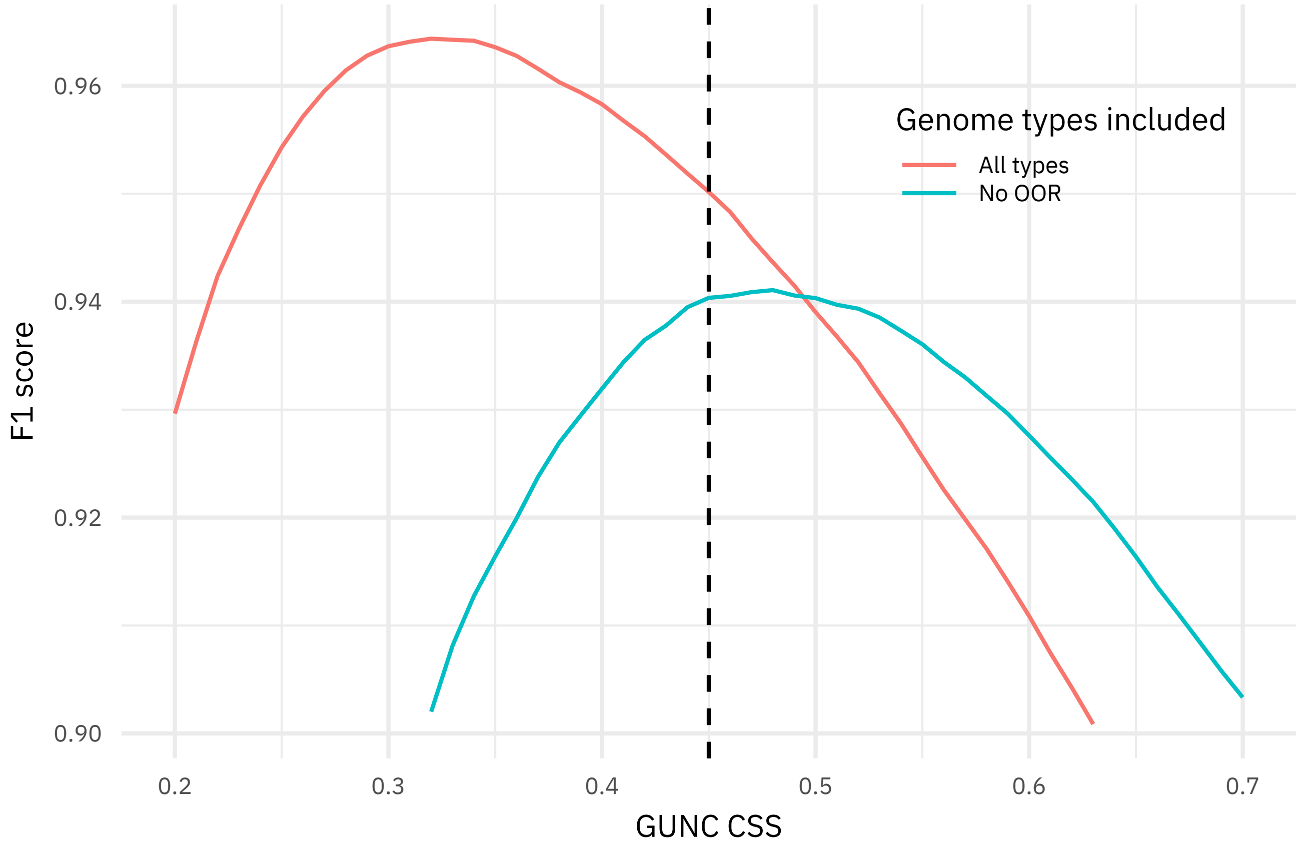

Fig. S12. Mean F-score of 10 iterations of 10,000 non-chimeric vs 10,000 chimeric genomes across different values of GUNC CSS cutoffs used to separate between chimeric and non-chimeric genomes. For “All types” genome types 1 and 2 are used as non-chimeric and types 3, 4 and 5a are used as chimeric (type 5b excluded since it is not expected to be detected at all). For “No OOR” genome type 1 only is used as non-chimeric and types 3 and 4 are used as chimeric. The cutoff with high performance at “No OOR” was chosen so that its performance is as high as possible in the “All types” setup without any significant loss to “No OOR” setup performance.
