# Supplementary material for: GUNC: detection of chimerism and contamination in prokaryotic genomes
Source: Genome Biol. 2021 Jun 13;22:178. doi: 10.1186/s13059-021-02393-0 (PMC8201837; doi:10.1186/s13059-021-02393-0)
Supplement: Supplementary file 3 — Additional file 3. Review history. [file 13059_2021_2393_MOESM3_ESM.docx]

**1^st^ round**

**Referee 1**

The authors presented GUNC, a bioinformatics tools for detecting chimerism and contaminations in prokaryotic genomes based on assessing taxonomic homogeneity of all genes found in the contigs of a MAG. This manuscript is concise and elegant. I enjoyed reading it. I think it will also be informative while not too technical the broader metagenomics community.

The major innovation of GUNC is the use of all genes. The authors insightfully identified the limitation of the current state-of-the-art: CheckM, that its "core" gene set is limited especially at deep branches. Instead, GUNC does not rely on the assumption of such core set, but integratively analyzes the taxonomic assignment of every single gene. I believe this is the correct direction. The metric based on the uncertainty coefficient is reasonable to me.

The benchmarks were well-designed. The defintions of seven types of contamination are very informative.

One valuable outcome of this work is the systematic examination of multiple popular genome databases and collection. It is interesting, though not surprising, to find out that a considerable proportion of reference genomes are chimeric. This resource is valuable to future metagenomics researchers.

The authors identified the purpose of GUNC: to complement, not to replace CheckM, as GUNC does not provide genome completeness information given the design. I think this is a reasonable suggestion.

However, I have the following major concerns:

I had problem installing GUNC. I did both "conda create -n gunc -c bioconda gunc python=3" attempting to create a dedicated environment for GUNC, and "conda install -c bioconda gunc" in an existing Python 3 environment. Both threw dependency conflict errors. I tested this on a desktop computer and a cluster server and got the same error. The developers of GUNC should work to resolve it.

The reference genome set was generated from proGenomes, and proGenomes is ranked the highest in the benchmarks shown in Fig. 3a. I question the fairness of this comparison. Can the authors apply the same reference-generating protocol on some other genome sets and run the benchmark again? How will the resulting ranking change?

Meanwhile, if I understand correctly, proGenomes uses the NCBI taxonomy, which is known to contain many consistencies when compared against the phylogeny. Have the authors tried the GTDB taxonomy?

The authors didn't explain how they ran CheckM to generate the benchmarks that were outcompeted by GUNC (Fig. 3) (or if they did and I overlooked in which case I apologize). I think one most important thing in this analysis is that CheckM relies on a database they generated back in 2015 (which hasn't been updated since then, which is unfortunate, but I may overlook). If the comparison is between GUNC + proGenomes 2.1 vs. CheckM + CheckM database (2015), then there is a strong expectation that the former will outperform the latter, not because of the superiority of the algorithm but because of the freshness and comprehensiveness of the database. The authors should clarify this point and remove the bias in the benchmark if not already done so.

Finally, one limitation of GUNC by design is that it still uses taxonomy as a proxy of phylogeny. This inherently means that the resolution of lineage placement is limited. If I understand correctly, the proGenomes database has some 80,000+ genomes, and they needed to be placed under the seven standard taxonomic ranks. I wouldn't suggest that this needs to be changed at this moment, but just wanted to point out a direction for future improvement.

**Referee 2**

In the present manuscript, the authors describe, benchmark and apply GUNC, a novel software for assessing contaminations in prokaryotic genomes. Overall, I find this study very appealing, it is certainly timely, and I consider it a relevant contribution to the field. It is for this reason, that I have mostly only minor comments, which should be straightforward to address. In general, I think it would be great if the authors could improve the clarity of the manuscript.

Major

1. In Figure S8, the authors show the results for Clostridium. The GUNC analysis indicates that the analyzed gene set of ~2,200 genes, which is in the range of the expected gene set size, represents two different species. If this assignment is correct, what is the likely scenario that results in the artificial fusion of two clostridium species where each species contributes about half of the gene set (C. butyricum has in fact a somewhat higher share)? Or is this an indication that assignments on the species level are simply tricky?

2. The GUNC taxonomic assignment is based on Diamond using the best hit as a tentative taxonomic assignment for a gene. What is the rationale to not instead apply an LCA algorithm, for example, to take assignment uncertainty into account?

Minor essential

1. Do the authors use contig and scaffold synonymously, or are scaffold broken up into the corresponding contigs?

2. A considerable effort was made by the authors to compare GUNC with MIMAG and CheckM. I think the manuscript would become a lot more accessible when both MIMAG and CheckM are thoroughly introduced, integrated with GUNC and the different concepts made clear. In fact, MIMAG appears to be mentioned first on page 11 within the results and is not covered in the introduction.

3. In the existing description of CheckM, it is unclear why a genomic chimera of unrelated lineages—by the way, a concept that does not exist if we believe in a tree of life—should lead to a taxonomic assignment closer to the root. Is this true, even for low levels of contamination?

4. P6, l58. The authors should re-consider the definition of redundant vs non-redundant contamination. In the current manuscript, foreign genes that extend a gene family are considered redundant contamination, and only those that seed a new gene family are considered non-redundant. This raises the question about a) what is the precise definition of a gene family here, and b) why would the addition of a very remotely related member of a gene family, which is otherwise not present in the genome, be a redundant contamination? In a BUSCO-world, I would consider a redundant contamination one that results in a 'duplicated BUSCO', whereas a non-redundant contamination either results in a BUSCO gene that is otherwise not present, are alternatively, replaces the original gene. If this description is revised, it would be good to carry this over to Page 10, 2nd par, where it is described how non-redundant contamination was simulated.

5. P7, l9. Coming back to non-redundant annotations: here it is stated that they add "something new". This is not necessarily the case if it is a replacement, I think.

6. P8, l55. What label is meant here exactly

7. P8, l59. What is the CSS value of a genome that is free of contaminations? Looking at the formula, this is also 1, correct? This should be mentioned here. In any case, the sentence 'The CSS quantifies the degree…' should better directly state something lik "low CSS values indicate… whereas high values…"

8. P9, line 21. The authors mention here the sensitivity of CSS. They may consider to refer here to a corresponding figure.

9. P9, line 24. GUNC appears to be sensitive when there contaminating sequences reside on their own contig. Does this imply that GUNC does not perform well when the genome sequence is represented by one scaffold?

10. P10, l32. The authors mention a training set, however, I do not see that anything is trained here.

11. Page 10, 2nd par. This paragraph is a mix of data set simulation and initial analysis. To improve clarity, it would be great to have the data sets described first, followed by the individual analysis steps.

12. P12, l22. 'shallower phylogenetic novelty' should be rephrased (see also Minor discretional comments, #7

13. P13, 2nd par. I think it is a great idea to assess the impact of HGT on the detection of contamination. Unfortunately, I found the corresponding paragraph quite confusing. I think the information provided should be disentangled, better explained, and the take home message should better stand out. Just to give two examples: The authors state that they found, as expected, a higher proportion of chimerism calls among the HGT genomes compared to proGenomes 2.1. This somehow implies that the authors expect that GUNC gets confused by HGT. However, at a later point, I somehow get a different impression. Moreover, 'well-defined' artefacts are mentioned as a reason why 407 HGT genomes are flagged by GUNC. The nature of the artefacts remains unclear.

14. The information on P16, 2nd par might be better presented in a table

15. P18, l9. The concept of redundant contamination between identical lineages should be explained. I am lost here.

16. P21, equation 3. please explain what n represents

17. P23, equation 2. The authors write in the context of RRS: "This signature is formalized as the term Portion_{genes retained}". However, I somehow missed the corresponding filtering step

Minor discretional comments

1. Title: I am wondering a bit about the difference of chimerism and contamination. I would think that one detects contaminations in the gene set and then concludes on chimerism.

2. P6, l18. Contamination of reagents or culture media will also contribute to MAG contamination. Although I do get that the authors talk about the principle error source, I would prefer if the additive character of error sources is mentioned. Alternatively, if the procedure to arrive at MAGs largely removes contaminations from reagents and media, then this would be nice to mention.

3. P6, 1st sentence. Check and consider rephrasing

4. P6, l38. BUSCO is mentioned in a context that generates the impression that it is genuinely used for contaminant detection. This, however, is not the case.

5. P6, l42. Type II errors are mentioned here. I do not think that outside a statistical testing context this makes much sense. Better to explain what you mean by that

6. The figures are overall nice. However, I find them a bit too crowded and the authors may consider to make them a bit more easily accessible. Moreover, the different shades of orange, violet and red are a bit hard to distinguish. It might help to use dashed line, crosses, and the like to facilitate an easier orientation in the plots.

7. P7, line 13. Probably find a better word for 'estimate' (maybe 'assess/identify'), and 'phylogenetically novel' reads strange. I suggest to omit this and just state "lineages that are not well represented…". In a similar context, I have the impression that using the term "phylogeny" in general does more harm than good (cf. p8, l37; P9, line 14, p12, l22), and I suggest to remove it.

8. P11, l18-28. It is not clear where to look at. Probably, the authors forgot to point to the corresponding supplementary figure.

9. P12, l18. The placeholder 'divergence' is not easy to interpret. I suggest to refer to the last common ancestor of two taxa, instead. LCA at genus, family or order level, is something that at least I could follow better.

**Author response**

**Reviewer #1**: The authors presented GUNC, a bioinformatics tools for detecting chimerism and contaminations in prokaryotic genomes based on assessing taxonomic homogeneity of all genes found in the contigs of a MAG. This manuscript is concise and elegant. I enjoyed reading it. I think it will also be informative while not too technical the broader metagenomics community.

The major innovation of GUNC is the use of all genes. The authors insightfully identified the limitation of the current state-of-the-art: CheckM, that its "core" gene set is limited especially at deep branches. Instead, GUNC does not rely on the assumption of such core set, but integratively analyzes the taxonomic assignment of every single gene. I believe this is the correct direction. The metric based on the uncertainty coefficient is reasonable to me.

The benchmarks were well-designed. The defintions of seven types of contamination are very informative.

One valuable outcome of this work is the systematic examination of multiple popular genome databases and collection. It is interesting, though not surprising, to find out that a considerable proportion of reference genomes are chimeric. This resource is valuable to future metagenomics researchers.

The authors identified the purpose of GUNC: to complement, not to replace CheckM, as GUNC does not provide genome completeness information given the design. I think this is a reasonable suggestion.

*We thank the reviewer for their positive feedback and for their constructive comments. Addressing the reviewer’s points has helped us to further support our findings and to clarify  their presentation in the manuscript.*

However, I have the following major concerns:

I had problem installing GUNC. I did both "conda create -n gunc -c bioconda gunc python=3" attempting to create a dedicated environment for GUNC, and "conda install -c bioconda gunc" in an existing Python 3 environment. Both threw dependency conflict errors. I tested this on a desktop computer and a cluster server and got the same error. The developers of GUNC should work to resolve it.

***RESPONSE:*** *We thank the reviewer for pointing this out and we apologize for this inconvenience. There was an issue for a time where conda selected an old version of GUNC which could cause problems like the one described, however this version has been labelled in bioconda and should no longer be installed. We have tested the conda installation on different systems but cannot test on all system variants, however, we are open to receiving issues on github to solve specific issues (see e.g.* [*https://github.com/grp-bork/gunc/issues/5*](https://github.com/grp-bork/gunc/issues/5)*). Alternatively, GUNC can be manually installed via pip and we additionally provide both docker and singularity containers which should avoid any system specific issues (https://grp-bork.embl-community.io/gunc/installation.html).*

The reference genome set was generated from proGenomes, and proGenomes is ranked the highest in the benchmarks shown in Fig. 3a. I question the fairness of this comparison. Can the authors apply the same reference-generating protocol on some other genome sets and run the benchmark again? How will the resulting ranking change?

***RESPONSE:*** *We agree with the reviewer that circular comparisons in benchmarks should be avoided as much as possible. For the comparisons in Fig 3 concerning proGenomes, we explicitly excluded self-hits for GUNC scoring in a leave-one-out setup, introducing a bias* ***against*** *proGenomes if anything. In Fig 3, three different proGenomes (PG)-derived genome sets are tested: PG2, PG2.1 and GUNC db. PG2 is the originally published PG set by Mende et al (PMID:28053165). PG2.1 is an updated version with some further filtering of genomes based on various criteria, including checkM. The GUNC db is derived from PG2.1 by taking exclusively species representative genomes. Even the GUNC db itself does not perform at 100% accuracy in Fig 3a which can be considered the tool’s (small) level of residual error. PG2, PG2.1 and GTDB are subsets of GenBank and RefSeq, filtered using different approaches. GTDB moreover contains MAGs, and as Fig 3a shows, GTDB-MAGs contain a higher fraction of chimeric genomes than GTDB isolate genomes. We generally interpret the observed differences between these databases in Fig 3a to be due to differences in genome filtering, not bias.*

*Nevertheless, in response to the reviewer’s comment we have now recomputed Fig. 3a using 31,910 species-representative genomes from the GTDB database with GTDB taxonomy as GUNC reference. This novel figure S9 is reproduced below for convenience. Using GTDB as reference db did not change the ranking of genome sets (see also next comment); however, run times and resource requirements were significantly increased.*

Meanwhile, if I understand correctly, proGenomes uses the NCBI taxonomy, which is known to contain many consistencies when compared against the phylogeny. Have the authors tried the GTDB taxonomy?

***RESPONSE:*** *We thank the reviewer for this suggestion! We have* *now analysed two additional types of re-simulated data: (i) using the GUNC reference db, but with GTDB taxonomy instead of NCBI taxonomy (results in new fig S4); (ii) using the GTDB with GTDB taxonomy as reference, as discussed in the previous comment (results in new fig S5). After confirming that our originally calibrated cutoffs for the GUNC clade separation score were valid in both these scenarios, we recomputed GUNC scores for all datasets to reproduce alternative versions of fig 2b-f and fig 3a.*

*In all re-simulated setups, use of the GTDB taxonomy either resulted in no significant change in accuracy, or indeed slightly worse performance. Moreover, the ranking of genome sets in fig S9 did not change, although more genomes were labelled as chimeric using the GTDB taxonomy, especially among MAGs. In response to the reviewer’s comment we now also provide a pre-formatted GTDB v95 as alternative GUNC database for download on the tool’s website. However, using this database entails a significant performance penalty (around 3x longer computation times as well as higher resource requirements), while not providing more accurate results, as outlined above.*

The authors didn't explain how they ran CheckM to generate the benchmarks that were outcompeted by GUNC (Fig. 3) (or if they did and I overlooked in which case I apologize). I think one most important thing in this analysis is that CheckM relies on a database they generated back in 2015 (which hasn't been updated since then, which is unfortunate, but I may overlook). If the comparison is between GUNC + proGenomes 2.1 vs. CheckM + CheckM database (2015), then there is a strong expectation that the former will outperform the latter, not because of the superiority of the algorithm but because of the freshness and comprehensiveness of the database. The authors should clarify this point and remove the bias in the benchmark if not already done so.

***RESPONSE:*** *We fully agree that ideally, all benchmarks should be run using identical references as input. Unfortunately, this is simply not possible for CheckM. As the reviewer points out, the CheckM db was last updated in 2015 and the tool does not provide the option to train or provide custom reference sets. Training a CheckM reference is expected to be non-trivial, as it entails the inference of a genome phylogeny, the semi-automated selection of marker gene sets and parameter tuning that cannot be automated. For leave-one-out validations as performed in some of our tests, this process would have to be repeated many times. This is simply not practicable and beyond the scope of our present study.*

*CheckM uses its underlying db differently than GUNC, so both tools are not directly comparable. CheckM starts with phylogenetic or taxonomic placement, so it interpolates its reference space by design; subsequent steps are then performed on marker gene sets inferred from this placement. GUNC, on the other hand, maps sequences directly to the db and processes hits. In this sense, the reference db (both for CheckM and GUNC) is essential to performance in different ways. In our benchmarks, we only compared CheckM and GUNC in simulation setups where it makes sense; in particular, since one cannot easily re-train CheckM references (see above), we could not include CheckM in true “out-of-reference” tests.*

Finally, one limitation of GUNC by design is that it still uses taxonomy as a proxy of phylogeny. This inherently means that the resolution of lineage placement is limited. If I understand correctly, the proGenomes database has some 80,000+ genomes, and they needed to be placed under the seven standard taxonomic ranks. I wouldn't suggest that this needs to be changed at this moment, but just wanted to point out a direction for future improvement.

***RESPONSE:*** *We fully agree with the reviewer that using a fully resolved, taxonomy-agnostic phylogeny as reference may further improve performance in principle. Indeed, we experimented with this approach during earlier stages of the project. The use of 7-level taxonomic classifications is of course only a proxy for phylogeny: it can be thought of as a heuristic. The main advantages of our approach are higher speed and limited resource requirements: a true phylogenetic placement for every individual gene would increase processing times 10-100x (depending on the method used) at a significantly higher memory footprint. Even placing genes into pre-computed gene families, as provided e.g. in the eggNOG or OMA databases, is significantly slower than our current approach. At the same time, we did not observe significantly higher accuracies when using phylogenetic or gene family placements. Even though the 7-level NCBI taxonomy is undeniably not optimal, it seems to provide a very good and performant heuristic to address our problem in practice.*

**Reviewer #2**: In the present manuscript, the authors describe, benchmark and apply GUNC, a novel software for assessing contaminations in prokaryotic genomes. Overall, I find this study very appealing, it is certainly timely, and I consider it a relevant contribution to the field. It is for this reason, that I have mostly only minor comments, which should be straightforward to address. In general, I think it would be great if the authors could improve the clarity of the manuscript.

*We thank the reviewer for their careful and diligent review, and the insightful and constructive comments. We found that we agreed with almost all of them, and implementing the reviewer’s suggestions has improved the manuscript considerably in our view.*

Major

1.   In Figure S8, the authors show the results for Clostridium. The GUNC analysis indicates that the analyzed gene set of ~2,200 genes, which is in the range of the expected gene set size, represents two different species. If this assignment is correct, what is the likely scenario that results in the artificial fusion of two clostridium species where each species contributes about half of the gene set (C. butyricum has in fact a somewhat higher share)? Or is this an indication that assignments on the species level are simply tricky?

***RESPONSE:*** *Figure S8 (now became S11) shows contig level classifications for one MAG generated by Almeida et al., 2019. It is an example of a common binning error we observed in other MAGs as well, in particular if the binning algorithm uses only local information (coverage only in the sample from which contigs were assembled, no co-abundance information across multiple samples). For this particular MAG, individual contigs are taxonomically ‘pure’, i.e. genes in one contig mapped to either C. perfringens or C. butyricum, but not to both within the same contig. This strongly suggests that the MAG is a chimera of two incomplete genomes. Although GUNC does not use species-level assignments by default, they are often sufficiently accurate in practice, as demonstrated in this example.*

*In response to this comment, we analysed this particular MAG further. CheckM estimates that it is 79.31% complete but only 1.72% contaminated, in line with our argument that CheckM may underestimate non-redundant contamination. We next screened the genome for the ‘specI’ set of 40 universal single copy marker genes as highly reliable taxonomic markers, detecting 13 in total. As shown in the below table, the taxonomic classification of these genes on each contig aligned perfectly with GUNC’s output. Only one MG (COG0495) was detected twice, with conflicting taxonomy. This pattern is in line both with CheckM’s low contamination estimate (very low redundancy among single copy marker genes) and GUNC’s high confidence chimerism call.*

*Universal_MG             Contig_ID                        MG_species_assignment        #contig_from_right*

*COG0012   NODE_28_length_161529_cov_188.159_31     Clostridium perfringens   2*

*COG0016   NODE_28_length_161529_cov_188.159_50     Clostridium perfringens   2*

*COG0052   NODE_48_length_119686_cov_179.945_79     Clostridium perfringens   3*

*COG0124   NODE_28_length_161529_cov_188.159_95     Clostridium perfringens   2*

*COG0172   NODE_43_length_126441_cov_179.412_100* ***Clostridium butyricum***  *15*

*COG0184   NODE_48_length_119686_cov_179.945_98     Clostridium perfringens    3*

*COG0215   NODE_198_length_26272_cov_188.295_25* ***Clostridium butyricum***  *26*

***COG0495*** *NODE_23_length_174748_cov_145.974_99* ***Clostridium butyricum***  *13*

***COG0495*** *NODE_47_length_120127_cov_178.613_49     Clostridium perfringens    5*

*COG0525   NODE_28_length_161529_cov_188.159_81     Clostridium perfringens    2*

*COG0533   NODE_276_length_11794_cov_166.313_2* ***Clostridium butyricum*** *41*

*COG0541   NODE_48_length_119686_cov_179.945_66     Clostridium perfringens    3*

*COG0552   NODE_48_length_119686_cov_179.945_64     Clostridium perfringens    3*

2.   The GUNC taxonomic assignment is based on Diamond using the best hit as a tentative taxonomic assignment for a gene. What is the rationale to not instead apply an LCA algorithm, for example, to take assignment uncertainty into account?

***RESPONSE:*** *We thank the reviewer for this suggestion. As outlined in our above response to a related suggestion on using full phylogenies by reviewer #1, we explored both phylogenetic placement (LCA) and the use of ortholog trees (via eggNOG) during earlier stages of the project. While we agree that LCA may be even more accurate in principle, it requires very high-quality gene trees, introduces a large penalty on computational speed and resource usage, even though we found no significant increase in accuracy in practice. We therefore consider the 7-level NCBI taxonomy a robust and performant heuristic in practice.*

Minor essential

1.   Do the authors use contig and scaffold synonymously, or are scaffold broken up into the corresponding contigs?

***RESPONSE:*** *We avoided the term ‘scaffold’ in the main text on purpose, as contig scaffolding is not expected to impact our results. For MAGs, contigs and scaffolds are usually the same, as most assemblers do not scaffold metagenomic contigs. For isolate genomes, we used scaffolds directly and did not break them down. As GUNC is gene-centric, results should not be affected either way, unless contigs from different sources were erroneously scaffolded (forming chimeric scaffolds).*

2.   A considerable effort was made by the authors to compare GUNC with MIMAG and CheckM. I think the manuscript would become a lot more accessible when both MIMAG and CheckM are thoroughly introduced, integrated with GUNC and the different concepts made clear. In fact, MIMAG appears to be mentioned first on page 11 within the results and is not covered in the introduction.

***RESPONSE:*** *We thank the reviewer for pointing this out. We have now stated more clearly in the respective section that MIMAG criteria are a combination of (CheckM’s) completeness and contamination criteria, not a separate method.*

3.   In the existing description of CheckM, it is unclear why a genomic chimera of unrelated lineages—by the way, a concept that does not exist if we believe in a tree of life—should lead to a taxonomic assignment closer to the root. Is this true, even for low levels of contamination?

***RESPONSE:*** *The reviewer is of course correct: there are no truly ‘unrelated’ lineages on a tree of life. We have now adjusted the phrasing to ‘not closely related’, ‘distantly related’ and ‘deeply branching’ where applicable.*

*The biases introduced by CheckM’s phylogenetic placement step were originally acknowledged and explored by the authors in the original CheckM publication (Parks et al, PMID:25977477; e.g., figure S8 and supplementary discussion). We have now also added a new figure S1 (reproduced below for convenience) to empirically quantify the described effect on CheckM placements. We simulated chimeric genomes as described in the main text, using source genomes that diverged at different taxonomic levels. We observed that the bias in CheckM’s lineage placements increased with increasing levels of simulated contamination, as expected. If two source genomes were mixed 1:1 (50% contamination), CheckM placed the resulting chimera at kingdom level in most cases, regardless of the true level of source genome divergence. Interestingly, placement bias was mostly driven by the degree of contamination (higher bias at higher contaminations), less by source genome relatedness. Indeed, for ‘deeper’ chimeras (e.g., of source genomes from different orders or classes) at lower contamination, placement bias was towards higher phylogenetic levels instead (placement at genus or family level). Both over-conservative and over-confident placements are expected to negatively affect the accuracy of CheckM estimates; we have adapted the phrasing in the main text accordingly*

4.   P6, l58. The authors should re-consider the definition of redundant vs non-redundant contamination. In the current manuscript, foreign genes that extend a gene family are considered redundant contamination, and only those that seed a new gene family are considered non-redundant. This raises the question about a) what is the precise definition of a gene family here, and b) why would the addition of a very remotely related member of a gene family, which is otherwise not present in the genome, be a redundant contamination? In a BUSCO-world, I would consider a redundant contamination one that results in a 'duplicated BUSCO', whereas a non-redundant contamination either results in a BUSCO gene that is otherwise not present, are alternatively, replaces the original gene. If this description is revised, it would be good to carry this over to Page 10, 2nd par, where it is described how non-redundant contamination was simulated.

***RESPONSE:*** *We thank the reviewer for pointing this out and we agree that the distinction between ‘redundant’ vs ‘non-redundant’ contamination is, to some extent, theoretical. However, as we argue in the manuscript, estimators of contamination have so far been biased towards detecting the ‘redundant’ type (‘duplicated BUSCO’ in the reviewer’s words, this reflects CheckM’s SCG-based approach to contamination estimation). We did not define relatedness ‘thresholds’ at which non-redundant contamination would become redundant, as this would depend on the granularity of observation, as the reviewer correctly points out: a gene (ortholog) may be ‘novel’ (from different groups) at family level, but in the same group at e.g. class level (i.e., ‘redundant’). Following the reviewer’s suggestion, we have now adapted the phrasing to reflect this, indicating that `redundant` contamination involves close(r) orthologs, whereas ‘non-redundant’ contamination are more distant orthologs.*

5.   P7, l9. Coming back to non-redundant annotations: here it is stated that they add "something new". This is not necessarily the case if it is a replacement, I think.

***RESPONSE:*** *With the adapted phrasing (see above), we hope that it is now more evident that we refer to ‘something new’ in the sense of more distantly related or entirely novel orthologs, reflecting the reviewer’s suggestion.*

6.   P8, l55. What label is meant here exactly

***RESPONSE:*** *‘Contig label’ referred to the contig assignment, i.e. contig ID. To clarify this, we have now changed the sentence to read, “expected entropy when there is no relationship between* ***taxonomic labels across contigs.****”*

7.   P8, l59. What is the CSS value of a genome that is free of contaminations? Looking at the formula, this is also 1, correct? This should be mentioned here. In any case, the sentence 'The CSS quantifies the degree…' should better directly state something lik "low CSS values indicate… whereas high values…"

***RESPONSE:*** *Thanks for pointing this out. We have now clarified that the CSS value is 0 when the genome is free of contamination as shown in the formula in P23, l20, with an additional pointer to the Methods.*

8.   P9, line 21. The authors mention here the sensitivity of CSS. They may consider to refer here to a corresponding figure.

***RESPONSE:*** *Fixed.*

9.   P9, line 24. GUNC appears to be sensitive when there contaminating sequences reside on their own contig. Does this imply that GUNC does not perform well when the genome sequence is represented by one scaffold?

***RESPONSE:*** *The reviewer is right: GUNC is not designed to detect contamination in this edge case. However, we do not think that this will impact performance in practice. Genomes in a single scaffold are usually the result of curation, or of very deep sequencing of an isolate. Contamination could be introduced then via mis-assembly, but this is not frequent in practice even for metagenomic assemblies, as discussed in the manuscript. Even though GUNC will not flag single-scaffold genomes as chimeric, it will still report if >2% of genes map to a distinct taxonomic group (a potential contaminant), and the locations of mis-matching genes will be available from its raw output.*

10.   P10, l32. The authors mention a training set, however, I do not see that anything is trained here.

***RESPONSE:*** *We have now adjusted the phrasing to “****reference*** *set”.*

11.   Page 10, 2nd par. This paragraph is a mix of data set simulation and initial analysis. To improve clarity, it would be great to have the data sets described first, followed by the individual analysis steps.

***RESPONSE:*** *In response we have now added a more explicit pointer to the Methods section which includes a detailed overview as suggested by the reviewer.*

12.   P12, l22. 'shallower phylogenetic novelty' should be rephrased (see also Minor discretional comments, #7

***RESPONSE:*** *We fully agree, thanks for catching this. Changed to ‘lower taxonomic divergence’ in the text.*

13.   P13, 2nd par. I think it is a great idea to assess the impact of HGT on the detection of contamination. Unfortunately, I found the corresponding paragraph quite confusing. I think the information provided should be disentangled, better explained, and the take home message should better stand out. Just to give two examples: The authors state that they found, as expected, a higher proportion of chimerism calls among the HGT genomes compared to proGenomes 2.1. This somehow implies that the authors expect that GUNC gets confused by HGT. However, at a later point, I somehow get a different impression. Moreover, 'well-defined' artefacts are mentioned as a reason why 407 HGT genomes are flagged by GUNC. The nature of the artefacts remains unclear.

***RESPONSE:*** *We thank the reviewer for pointing this out. Among 3,465 predicted HGT genomes, GUNC flagged 407 (11.7%) as chimeric. Fig S7 (previously S4) provides a breakdown on these 407 genomes. We have now revised the paragraph to add another pointer to Fig S7 and to clarify that the ‘well-defined’ artifacts are the discussed 71 Salmonella-Klebsiella genome pairs where GUNC’s incorrect call can be explained by a database error (failure to automatically remove a compatible plasmid sequence). So these 71 genomes (out of 407 GUNC-flagged HGT genomes) are false positive chimera calls, but traceable to a very specific effect (hence, ‘well-defined’). At least 120/407 flagged genomes were true positive chimera calls: 34 were confirmed manually, and another 86 were putative donor genomes in an HGT event for which GUNC detected contamination elsewhere in the genome.*

14.   The information on P16, 2nd par might be better presented in a table

***RESPONSE:*** *Sets from GMGC have been annotated with their names from Fig3a for clarity. We have also added additional references to Fig. 3e on which the discussion is based.*

15.   P18, l9. The concept of redundant contamination between identical lineages should be explained. I am lost here.

***RESPONSE:*** *By design, GUNC is ‘blind’ to duplications within a genome. For example, if one genome were (artificially) fully duplicated, CheckM would correctly estimate it as being 100% ‘contaminated’ (or 100% redundant). GUNC, in contrast, would not detect any contamination, as all contigs would be taxonomically pure, with no conflicting assignments. This is arguably a theoretical edge case, but we felt it was important to raise this limitation in the discussion. We have now adjusted the phrasing to clarify.*

16.   P21, equation 3. please explain what n represents

***RESPONSE:*** *Thanks for pointing this out! We have now corrected this error (‘n’ should have been ‘N’)..*

17.   P23, equation 2. The authors write in the context of RRS: "This signature is formalized as the term Portion_{genes retained}". However, I somehow missed the corresponding filtering step

***RESPONSE:*** *We have now clarified in the text that this refers to filtering against mapping noise by removing minority labels recruiting <2% of genes in the genome, as discussed earlier in the Methods; we agree that this context was missing.*

Minor discretional comments

1.   Title: I am wondering a bit about the difference of chimerism and contamination. I would think that one detects contaminations in the gene set and then concludes on chimerism.

*The terms ‘chimerism’ and ‘contamination’ of genomes are, unfortunately, not used consistently within the field. We fully agree with the reviewer’s point and indeed discussed this among the author team prior to the original submission: contamination can be measured to conclude on chimerism. However, ‘contamination’ is also often used in the field to describe genome errors that are not in line with ‘chimerism’ – e.g., duplications and other types of ‘redundancy’. Among others, Murat Eren wrote an insightful blog post on this, in particular on the (imprecise) usage of the terms ‘redundancy’ and ‘contamination’ (*[*https://merenlab.org/2016/06/09/assessing-completion-and-contamination-of-MAGs/*](https://merenlab.org/2016/06/09/assessing-completion-and-contamination-of-MAGs/)*). In view of this, we feel that the current title best describes what GUNC does, using common (though not 100% formal) terms in the field.*

2.   P6, l18. Contamination of reagents or culture media will also contribute to MAG contamination. Although I do get that the authors talk about the principle error source, I would prefer if the additive character of error sources is mentioned. Alternatively, if the procedure to arrive at MAGs largely removes contaminations from reagents and media, then this would be nice to mention.

*We agree that reagent/media/kit contaminants can affect MAGs as well. In practice, however, data handling and processing used to obtain metagenomes renders MAGs much less susceptible to these errors than isolate genomes. In vitro contaminant sources would mostly impact MAGs from low-microbial-biomass metagenomes, but e.g. for fecal samples, native microbial DNA would always outweigh contaminant DNA manyfold. We have now added this caveat to the text.*

3.   P6, 1st sentence. Check and consider rephrasing.

*Thanks for spotting this, we have now fixed the grammar.*

4.   P6, l38. BUSCO is mentioned in a context that generates the impression that it is genuinely used for contaminant detection. This, however, is not the case.

*The reviewer is of course right: BUSCO is designed to quantify genome completeness, not contamination, though duplications of BUSCO genes are not infrequently used to assess contamination downstream of running the tool. We have now rephrased that sentence, indicating that the* ***marker genes*** *provided by BUSCO, CheckM and other sources are used for these purposes.*

5.   P6, l42. Type II errors are mentioned here. I do not think that outside a statistical testing context this makes much sense. Better to explain what you mean by that

*Point well taken, this has been fixed.*

6.   The figures are overall nice. However, I find them a bit too crowded and the authors may consider to make them a bit more easily accessible. Moreover, the different shades of orange, violet and red are a bit hard to distinguish. It might help to use dashed line, crosses, and the like to facilitate an easier orientation in the plots.

*We thank the reviewer for the suggestion. The aim in picking colours was to visually connect related datasets (e.g., using different shades of the same colour for CheckM with different parameters). The sheer amount of data series makes it difficult to select a perfect palette; we based our choice of colours on the guides suggested on https://colorbrewer2.org/ .*

7.   P7, line 13. Probably find a better word for 'estimate' (maybe 'assess/identify'), and 'phylogenetically novel' reads strange. I suggest to omit this and just state "lineages that are not well represented…". In a similar context, I have the impression that using the term "phylogeny" in general does more harm than good (cf. p8, l37; P9, line 14, p12, l22), and I suggest to remove it.

*We thank the reviewer for spotting this inaccuracy and have rephrased accordingly.*

8.   P11, l18-28. It is not clear where to look at. Probably, the authors forgot to point to the corresponding supplementary figure.

*Fixed.*

9.   P12, l18. The placeholder 'divergence' is not easy to interpret. I suggest to refer to the last common ancestor of two taxa, instead. LCA at genus, family or order level, is something that at least I could follow better.

*While we agree that LCA is an established concept in the field, it implies a complete underlying phylogeny and properly resolved lineages. We feel that this would be misleading in our case, as we use taxonomic levels as containers for many lineages, their hierarchy only a proxy for the true underlying phylogeny. We therefore believe that ‘divergence level’ is a less loaded term.*

**2^nd^ round**

**Referee 1**

The authors have addressed all the comments made by me. I see that the manuscript has been significantly improved. I appreciate that the authors added the benchmark on the GTDB database, the result of which appear compelling. Providing the GUNC/GTDB database will also greatly facilitate the community. I agree with the authors that building an updated CheckM database is too difficult, and this missing part can be execused. I also agree with the authors' discussion of phylogeny vs. taxonomy. In conclusion, I think the current form of the manuscript is suitable for publication in Genome Biology.

Beyond, I want to make a few more comments on the software engineering. I followed the new instructions for installing GUNC. Again, I wasn't able to install using "conda install -c bioconda", but I was able to install using "pip install gunc". Previously, I also encountered multiple issues with conda, and it is much desired that the conda team (not the GUNC team) improve their product. The version and dependency of DIAMOND is another factor that frequently breaks things (in my experience).

Other than this, GUNC is a high-quality bioinformatic software tool. The command-line interface, the documentation, and the organization of code are all consistent with the modern practice of software engineering. Besides, as a new product from a prominent research team, the continuous maintenance and improvement of the codebase is anticipated. These should enhance the acceptance of this tool in the research community.

**Referee 2**

I think the authors did a very nice job in addressing the individual issues raised by reviewer 1 and myself, and I have only two very minor things remaining. I leave it to the discretion of the authors to follow my suggestion, or to stick with the original phrasing:

1. They write "close orthologs from gene families already present in the focal genome.". The term ‘from gene families’ is a bit confusing here. Why not just writing ‘close orthologs to genes genuinely present in the focal genome’

2. Consider replacing the phrase ‘deeply branching’ in “as a result of horizontal transfer between deeply branching lineages” with ‘evolutionarily distantly related’.
